# Pediatric de novo movement disorders and ataxia in the context of SARS-CoV-2

**DOI:** 10.1007/s00415-023-11853-5

**Published:** 2023-07-29

**Authors:** Nina-Maria Wilpert, Ana Luísa de Almeida Marcelino, Ellen Knierim, Pasquale Incoronato, Elisa Sanchez-Sendin, Olga Staudacher, Anne Drenckhahn, Petra Bittigau, Jakob Kreye, Harald Prüss, Markus Schuelke, Andrea A. Kühn, Angela M. Kaindl, Marc Nikolaus

**Affiliations:** 1 Department of Neuropediatrics, Charité-Universitätsmedizin Berlin, Corporate Member of Freie Universität Berlin, Humboldt-Universität zu Berlin, and Berlin Institute of Health (BIH), Campus Virchow-Klinikum, Augustenburger Platz 1, 13353 Berlin, Germany; 2 Center for Chronically Sick Children, Charité-Universitätsmedizin Berlin, Corporate Member of Freie Universität Berlin, Humboldt-Universität zu Berlin, and Berlin Institute of Health (BIH), Berlin, Germany; 3 Department of Neurology with Experimental Neurology, Movement Disorders and Neuromodulation Unit, Charité-Universitätsmedizin Berlin, Corporate Member of Freie Universität Berlin, Humboldt-Universität zu Berlin, and Berlin Institute of Health (BIH), Berlin, Germany; 4 German Center for Neurodegenerative Diseases (DZNE), Charité-Universitätsmedizin Berlin, Corporate Member of Freie Universität Berlin, Humboldt-Universität zu Berlin, and Berlin Institute of Health (BIH), Berlin, Germany; 5 Department of Neurology and Experimental Neurology, Charité-Universitätsmedizin Berlin, Corporate Member of Freie Universität Berlin, Humboldt-Universität zu Berlin, and Berlin Institute of Health (BIH), Berlin, Germany; 6 Department of Pediatric Respiratory Medicine, Immunology and Critical Care Medicine, Charité-Universitätsmedizin Berlin, Corporate Member of Freie Universität Berlin, Humboldt-Universität zu Berlin, and Berlin Institute of Health (BIH), Berlin, Germany; 7 NeuroCure Clinical Research Center, Berlin, Germany; 8 Department of Immunology, Labor Berlin GmbH, Berlin, Germany

**Keywords:** Pediatric movement disorder, Acute cerebellar ataxia, Acute cerebellitis, COVID-19, Neuroimmunology, Pediatric neurology

## Abstract

**Objective:**

In the fourth year of the COVID-19 pandemic, mortality rates decreased, but the risk of neuropsychiatric disorders remained the same, with a prevalence of 3.8% of pediatric cases, including movement disorders (MD) and ataxia.

**Methods:**

In this study, we report on a 10-year-old girl with hemichorea after SARS-CoV-2 infection and immunostained murine brain with patient CSF to identify intrathecal antibodies. Additionally, we conducted a scoping review of children with MD and ataxia after SARS-CoV-2 infection.

**Results:**

We detected antibodies in the patient's CSF binding unknown antigens in murine basal ganglia. The child received immunosuppression and recovered completely. In a scoping review, we identified further 32 children with de novo MD or ataxia after COVID-19. While in a minority of cases, MD or ataxia were a symptom of known clinical entities (e.g. ADEM, Sydenham's chorea), in most children, the etiology was suspected to be of autoimmune origin without further assigned diagnosis. (i) Children either presented with ataxia (79%), but different from the well-known postinfectious acute cerebellar ataxia (older age, less favorable outcome, or (ii) had hypo-/hyperkinetic MD (21%), which were choreatic in most cases. Besides 14% of spontaneous recovery, immunosuppression was necessary in 79%. Approximately one third of children only partially recovered.

**Conclusions:**

Infection with SARS-CoV-2 can trigger de novo MD in children. Most patients showed COVID-19-associated-ataxia and fewer-chorea. Our data suggest that patients benefit from immunosuppression, especially steroids. Despite treatment, one third of patients recovered only partially, which makes up an increasing cohort with neurological sequelae.

**Supplementary Information:**

The online version contains supplementary material available at 10.1007/s00415-023-11853-5.

## Introduction

After its outbreak in December 2019 in Wuhan, China [1], the Coronavirus SARS-CoV-2 (severe acute respiratory syndrome coronavirus type 2) spread rapidly around the world. By February 2022, more than 14 million children had been tested positive for COVID-19 [2] and the pediatric infection-induced SARS-CoV-2 sero-prevalence was around 70% in the United States [3]. Governments were forced to impose restrictions (e.g. school closure and social distancing) to fight exponential spread and hospital admissions. With unprecedented speed, vaccinations were developed and approved under an emergency use authorization by the FDA (Food and Drug Administration) and EMA (European Medicines Agency) in 2021, even for children.

Three years into the pandemic, we know that children present with milder respiratory symptoms than adults, possibly due to a stronger early innate antiviral response against the SARS-CoV-2 infection. [4] However, in addition to the general symptoms of COVID-19, rare neurologic abnormalities have been gradually reported with involvement of the central and peripheral nervous system, including hypo- and hyperkinetic movement disorders, as well as cerebellar and pyramidal signs [5–7]. Movement disorders, although often not life threatening, are functionally debilitating, stigmatizing, and pose a tremendous burden on the affected children and their families. The rising incidence of functional movement disorders during the COVID-19 pandemic was recently linked to the “Charcot’s Era at the Salpêtrière” and discussed in the scope of governments’ measures and their profound psychological impact [8–11]. Therefore, it is even more important to characterize and identify the non-functional de novo movement disorders associated with COVID-19. There is increasing evidence that such dysfunctions may have an autoimmune underpinning, thereby offering options for causative treatment [6, 12–14]. In fact, the SARS-CoV-2 virus is rarely detected in the cerebrospinal fluid (CSF), at least in adult patients [15], and neuropathological analyses suggest that cellular and humoral mechanisms rather trigger neuronal dysfunction [13, 16]. As examples, in children, post-infectious conditions with movement disorders (e.g. acute cerebellar ataxia or Sydenham’s chorea) with favorable outcomes have been well described [17, 18]. While single case reports and series of pediatric patients with de novo movement disorders in the context of a SARS-CoV-2 infection have been published, an overview of associated clinical phenotypes and their frequencies was still missing.

Stimulated by the here reported 10-year-old girl with hemichorea after SARS-CoV-2 infection/vaccination, we aim to provide an overview about the phenotypic spectrum, and diagnostic and therapeutic workup of all cases described until May 2022.

## Methods

### Search strategy

A scoping review was performed in MEDLINE (PubMed) using the search terms [“parkinsonism” OR “ataxia” OR “myoclonus” OR “tremor” OR “dystonia” OR “chorea” OR “movement disorders”] AND [“SARS-CoV-2” OR “COVID-19”], up to 24th May 2022. In addition, we filtered references of publications for relevant studies. Titles and abstracts were screened by one investigator for eligibility.

### Study selection criteria

Inclusion and exclusion criteria were pre-specified (Table 1). Criteria were applied to full-text articles by a first investigator. A second investigator was consulted in cases of uncertainty. Studies meeting the criteria were included into the qualitative analysis.

**Table 1:** Inclusion and exclusion criteria of the scoping review

	Inclusion	Exclusion
Patients’ age	< 18 years	≥ 18 years
COVID-19 infection/vaccination	Confirmed	No information
Data reported	De novo MD	Functional or hereditary MD
	Full text	Abstracts only
	In English	Other language
	Up to 24th May 2022	Beyond 24th May 2022
Type of publication	Original articles	Review articles
	Case reports	Expert opinions
	Brief reports	Conference proceedings

Children or adolescents either with a confirmed COVID-19 infection or COVID-19 vaccination and de novo movement disorders or ataxia were included into the study

*MD* movement disorder, *COVID-19* coronavirus disease 2019

### Data synthesis

Patients who (1) either had a history of SARS-CoV-2 infection or (2) were vaccinated against SARS-CoV-2 and were below the age of 18 years were included. The following patient data were extracted: age, sex, time from infection/vaccination to movement disorder, Multisystem Inflammatory Syndrome in Children (MIS-C) criteria [19], hypo-/hyperkinetic movement disorders, cerebellar and pyramidal signs, other neurologic/neuropsychiatric symptoms, general symptoms of COVID-19 on admission, therapy for movement disorders, outcome, relapses, observation time, comorbidities, laboratory findings in sera and CSF, neuroimaging, EEG. The data were summarized descriptively.

### Statistical analysis

In this retrospective study, no blinding, randomization, or formal power calculations were possible. We used descriptive statistics to present qualitative data as percentages of reported cases, quantitative data as median and range. For group comparisons, we applied the unpaired Student’s *t* test for continuous variables, with Welch’s correction due to unequal samples sizes, and the Fisher’s exact test for categorical variables. Two-tailed *p* values < 0.05 were considered significant. Analyses were carried out with GraphPad Prism (version 9.3.1).

### Ethics

For the case report, written informed consent for publication of individual images, videos and clinical details was obtained from the patient’s caregivers. Ethical approval for the study was obtained from the Institutional Review Board of Charité (EA2/121/17). The scoping review was performed retrospectively on published data which did not require an IRB vote.

## Results

### Case report

A 10-year old, previously healthy girl was admitted to our pediatric emergency department in February 2022 with striking left-sided chorea of the upper and lower limbs (Video 1, Supplementary methods 1). Hyperkinetic movements had evolved 2 weeks after her first SARS-CoV-2 infection with mild respiratory symptoms and four weeks after her first Comirnaty® vaccination. Choreatic movements were initially task-specific but occurred also at rest in the further course. Activities of daily living (e.g., dressing, hygiene) could hardly be performed independently. The right-handed patient preferred to fixate the left extremities with compensatory movements. In addition to hemichorea, the parents had noticed dysarthria as well as impulsive, irritable behavior, and attention deficit. Holocephalic headache had been reported weekly.

**Fig. 1:**
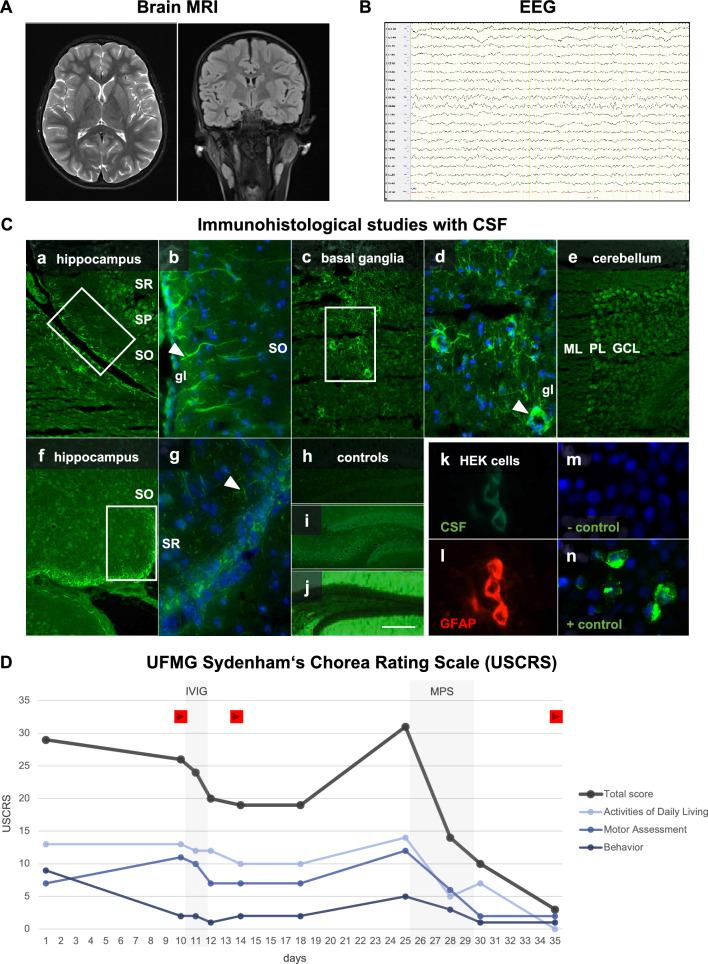
Clinical course of a ten-year-old girl with hemichorea associated to SARS-CoV-2.** A** Normal brain MRI T2 axial/coronal section and **B** EEG. **C**; **a**–**j** Immunohistochemistry using CSF and serum via tissue-based assay on fresh-frozen murine brain according to established protocols [39, 47]. **C**; **a**–**g** Immunofluorescence analyses on fresh-frozen murine brain revealed a positive signal of predominantly glial pattern labelling the fine cellular processes of astrocytes. **C**; **a**, **b** It was detected within the *glia limitans* in proximity to the *hippocampus*, **C**; **c**, **d** around large vessels across the basal ganglia, **C**; **d**, **e** and to lesser extent within the *cerebellum*. **C**; **f**, **g** Incubation with patient serum revealed a signal of similar distribution but considerably lower intensity **C**; **h**, **m** not seen in the respective negative, **C**; **j**, **n** background, **C**; **i** and positive control. The exact intrathecal autoantibodies could not be identified, neither in extended commercial panel analysis (EUROIMMUN) nor **C**, **k**, **l** via a cell-based approach with no clear binding to GFAP-expressing HEK293T-cells, when compared to a commercial anti-GFAP antibody. **D** The severity of chorea was assessed with the UFMG Sydenham’s Chorea Rating Scale (USCRS) [48] before/after IVIG, high-dose intravenous MPS. Red play buttons indicate the time of video recordings. *CSF *cerebrospinal fluid, *GFAP *glial fibrillary acidic protein, *GCL *granular cell layer, *gl glia limitans*, *IVIG *intravenous immunoglobulins, *ML *molecular layer, *MPS *methylprednisolone, *PL *Purkinje cell layer, *SO Stratum oriens*, *SP Stratum pyramidale*, *SR Stratum radiatum*. Size bars: 200 μm (**C**; **h**, **i**, **j**), 100 μm (**C**; **a**, **c**, **e**, **f**), 20 μm (**C**; **b**, **d**, **g**, **k**–**n**)

At time of admission, a SARS-CoV-2 RT-qPCR by nasopharyngeal swab was negative, and the patient did not suffer from general symptoms of COVID-19. Whereas an EEG, brain MRI, and exome sequencing results were normal, a lumbar puncture revealed slightly elevated protein levels with 0.5 g/l (normal range 0.15–0.45 g/l) and an increased opening pressure of 27 cm H_2_O (normal range ≤ 25 cm H_2_O) (Fig. 1). Consistent with elevated intracranial pressure, the fundoscopy revealed papilledema. Consequently, treatment with acetazolamide was initiated. CSF analyses including the white blood cell count, glucose, lactate, oligoclonal bands, SARS-CoV-2 serology were unremarkable and anti-neuronal/glial antibodies to established autoantigens were not detected. Immunofluorescence analyses on fresh-frozen murine brain revealed a positive signal of predominantly glial pattern labelling the fine cellular processes of astrocytes (Fig. 1). It was detected within the *glia limitans* in proximity to the *hippocampus*, around large vessels across the basal ganglia, and to lesser extent within the *cerebellum*. The findings did not resemble a neuropil staining pattern, as commonly detected in some forms of autoimmune encephalitis. Instead, they indicated a reactivity typical for acidic glial fibrillary acidic protein (GFAP), which would be characteristic for an astrocytopathy. However, the exact intrathecal autoantibodies could not be identified, neither in extended commercial panel analysis (EUROIMMUN) nor via a cell-based approach with no clear binding to GFAP-expressing HEK293T-cells, when compared to a commercial anti-GFAP antibody. Further investigations with CSF could not be carried out due to limited availability of CSF samples. Broad laboratory testing including TSH (thyroid stimulating hormone), electrolytes, antistreptolysin O titer, bacterial/viral PCR/serology, ceruloplasmin and vanillylmandelic acid, revealed no evidence for other causes of hyperkinetic movement disorders.

With the suspected diagnosis of autoantibody-mediated hemichorea, we initiated an immunosuppressive therapy. With no systematic collection of other cases available, we started treatment with IVIG (1000 mg/kg/day) for 2 days, which did not result in any significant improvement (Video 2, Fig. 1) over the following two weeks. High-dose methylprednisolone treatment (20 mg/kg/day) for 5 days was initiated thereafter, and symptoms resolved promptly (Video 3, Fig. 1). At 5 months follow-up visit, the movement disorder remained absent, but the patient continued to suffer from headaches without any further verified SARS-CoV-2 infection or a second SARS-CoV-2 vaccination. The papilledema had completely resolved at that time and brain MRI continued to be normal. Due to suspected side effects of acetazolamide, the medication was stopped, and the headache subsided completely. No relapse of movement disorders or other neuropsychiatric symptoms occurred within 6 months of observation time.

### Scoping review

Stimulated by the above-mentioned course of disease, we addressed the following questions: (1) Which phenomenology of movement disorders, cerebellar or pyramidal involvement evolves in the context of SARS-CoV-2? (2) What is the best diagnostic work-up and which clinical findings were made in other cases? (3) Could autoantibodies be identified in this cohort? (4) What would be the best treatment option? (5) How is the outcome and are relapses described? (6) Are de novo movement disorders, cerebellar or pyramidal signs an often-occurring side effect of the vaccination?

**Fig. 2:**
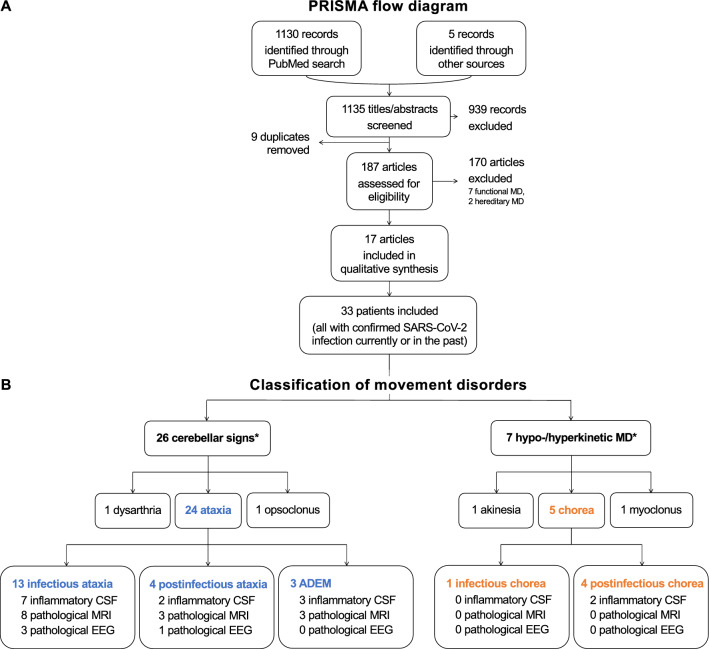
PRISMA flow diagram, study profile and classification of ataxia and movement disorders. After using the search terms [“parkinsonism” OR “ataxia” OR “myoclonus” OR “tremor” OR “dystonia” OR “chorea” OR “movement disorders”] AND [“SARS-CoV-2” OR “COVID-19”], up to 24^th^ May 2022 in MEDLINE (PubMed) 33 patients were included into the scoping review and classified according to their predominant phenomenology. *CSF* cerebrospinal fluid, *MD *movement disorder. *Predominant symptom

To offer some answers for pediatricians who see children with de novo movement disorders in the context of COVID-19, we performed a scoping review. Applying specific search terms on MEDLINE (see “Search strategy”), 1130 records could be identified according to the Preferred Reporting for Systematic Review and Meta-Analysis (PRISMA) consensus statement shown in Fig. 2. Five additional studies were found by screening references of the relevant literature. Thus, 1135 titles and abstracts were screened for eligibility. After excluding nine duplicates and another 939 studies not dealing with pediatric movement disorders, cerebellar or pyramidal signs in the context of COVID-19 infection/vaccination, the inclusion and exclusion criteria were applied on 187 full-text publications (Table 1). 170 studies were excluded, among them seven articles reporting on functional movement disorders and two on preexisting, hereditary syndromes with movement disorders. Finally, 17 articles were included for our analysis [5, 6, 20–34].

#### Study group

32 pediatric patients with de novo movement disorders, cerebellar or pyramidal signs were described in 17 articles. Patient-specific data of published cases including our case (*n* = 33) on the clinical presentation, diagnostic results, treatment and outcome are presented in Table 2 and Supplementary table 1. If data were not attributed to single patients [5], we report it specifically. A synopsis of all patients is summarized in Table 3. The cohort included infants to adolescents. The median age was 10 years (range 0.1–16.0 years). Sex was indicated in 30 of 33 cases and showed a balanced distribution with 15 female and 15 male patients. All patients had a positive SARS-CoV-2 RT-qPCR in the past medical history or on admission (Table 1). No child apart from the index case of this study had received the Comirnaty^®^ vaccination or another vaccination against SARS-CoV-2. Comorbidities were reported in the past medical history in only four patients and comprised cytothrombopenic purpura [6], type 1 diabetes mellitus, nephrolithiasis [28], anxiety, motor tics [23], and Sydenham’s chorea. [34]

**Table 2:**
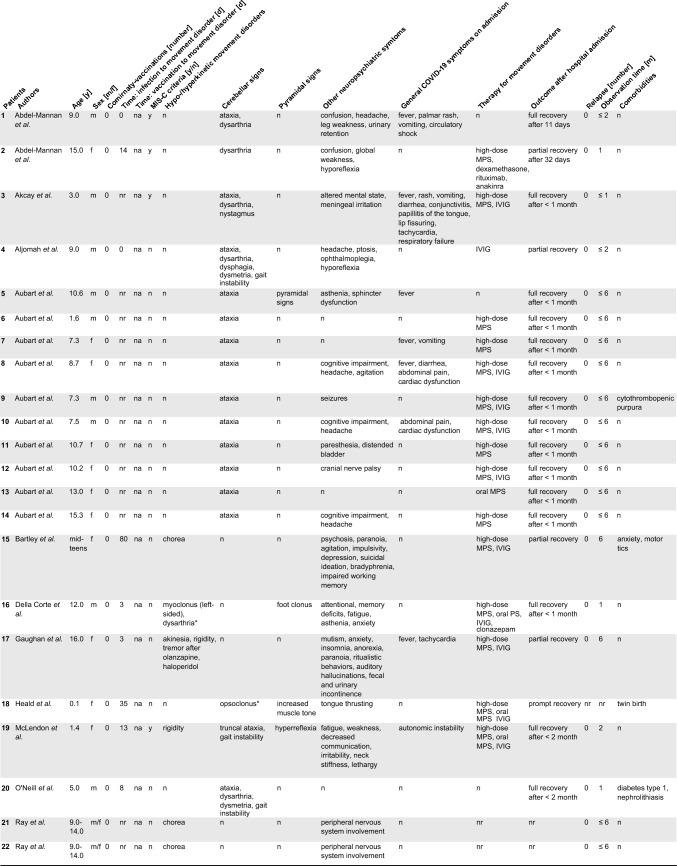

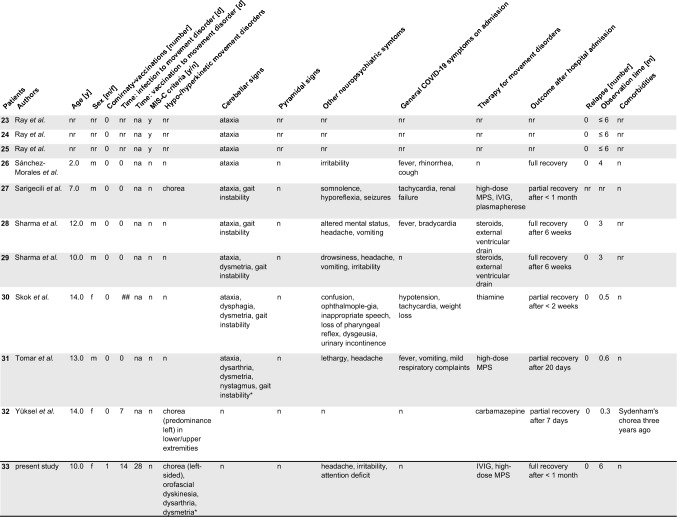
Patient-specific data of COVID-19 associated pediatric de novo movement disorders and ataxia

*nr* not reported, *na* not applicable, *np* not performed, *MPS* methylprednisolone, *IVIG* intravenous immunoglobulins, *y* yes, *n* no, *m* male, *f *female

^*^Videos online

**Table 3:** Clinical features of study group and comparison of COVID-19-ataxia/cerebellitis versus COVID-19-movement disorders

	Study group	COVID-19-cerebellar signs	COVID-19-MD	*p* values
	(*n* = 33)	(*n* = 26)	(*n* = 7)	
Age, years	10 (0.1–16.0), *n* = 29	9 (0.1–15.3), *n* = 23	13 (9.0–16.0), *n* = 6	**0.0127**
Sex				
Female	14/28 (50%)	10/23 (43%)	4/5 (80%)	0.3259
Male	14/28 (50%)	13/23 (57%)	1/5 (20%)	
Past medical history				
SARS-CoV-2 infection currently or in past	33/33 (100%)	26/26 (100%)	7/7 (100%)	> 0.9999
SARS-CoV-2 vaccination	1/33 (3%)	0/26 (0%)	1/7 (14%)	0.2121
Neurological/psychiatric comorbidities	2/28 (7%)	0/23 (0%)	2/5 (40%)	**0.0265**
Other comorbidities	2/28 (7%)	2/23 (9%)	0/5 (0%)	> 0.9999
Clinical features				
Infection to movement disorders time (days)	3 (0–280), *n* = 16	0 (0–280), *n* = 11	3 (3–80), *n* = 5	0.7803
Vaccination to movement disorders time (days)	28 (28), *n* = 1	na	28 (28), *n* = 1	na
Hypo-/hyperkinetic movement disorder^a^	7/33 (21%)	na	na	na
Cerebellar signs^a^	26/33 (79%)	na	na	na
Pyramidal signs^a^	0/33 (0%)	na	na	na
Neurological/psychiatric symptoms	25/30 (83%)	19/23 (83%)	6/7 (86%)	> 0.9999
Internistic symptoms on admission	13/30 (43%)	12/23 (52%)	1/7 (14%)	0.1038
MIS-C criteria	7/33 (21%)	7/26 (27%)	0/7 (0%)	0.2994
Investigations				
SARS-CoV-2 PCR positive on admission	19/27 (70%)	16/20 (80%)	3/7 (43%)	0.1445
SARS-CoV-2 seroconversion	8/27 (30%)	4/20 (20%)	4/7 (57%)	0.1445
Inflammatory CSF^b^	16/25 (64%)	13/21 (62%)	3/4 (75%)	> 0.9999
CSF proteine level (g/l)	0.36 (0.08–19), *n* = 23	0.59 (0.08–19)	0.48 (0.43–0.5)	0.3614
CSF white blood cell count (/µl)	4.5 (0–2000), * n* = 22	5 (0–2000)	1 (1–2)	0.2048
CSF autoantibody panel positive	3/10 (30%)	3/8 (38%)	0/2 (0%)	> 0.9999
CSF IF on murine brain sections positive	2/2 (100%)	0/0 (0%)	2/2 (100%)	> 0.9999
CSF SARS-CoV-2 PCR positive	0/8 (0%)	0/7 (0%)	0/1 (0%)	> 0.9999
CSF SARS-CoV-2 serology positive	2/3 (67%)	1/1 (100%)	1/2 (50%)	> 0.9999
Abnormal neuroimaging	16/30 (53%)	15/23 (65%)	1/7 (14%)	**0.0019**
Abnormal EEG	6/11 (55%)	4/4 (100%)	0/7 (0%)	**0.0030**
Treatment				
No treatment	4/28 (14%)	4/23 (17%)	0/5 (0%)	> 0.9999
Immunomodulation	22/28 (79%)	18/23 (78%)	4/5 (80%)	> 0.9999
Other treatment	2/28 (7%)	1/23 (4%)	1/5 (20%)	0.3307
Outcome				
Observation time (months)	2 (0.25–6), *n* = 31			
Spontaneous recovery	4/28 (14%)	4/23 (17%)	0/5 (0%)	> 0.9999
Full recovery after treatment	16/28 (57%)	14/23 (61%)	2/5 (40%)	0.6239
Partial recovery after treatment	8/28 (29%)	5/23 (22%)	3/5 (60%)	0.1231
Death	0/33 (0%)	na	na	na
Relapse	0/33 (0%)	na	na	na

Data are shown as *n*/reported items (%) or median (range), *n*

*MIS-C* Multisystem Inflammatory Syndrome in Children, *IF* immunofluorescence, *CSF* cerebrospinal fluid, *EEG* electroencephalogram, *MRI* magnetic resonance imaging, *PCR* polymerase chain reaction, *na* not applicable

^a^Predominant symptom

^b^Pleocytosis or elevated protein level or positive oligoclonal bands or CSF autoantibodies or positive immunofluorescence on murine brain sections

Two-tailed *p* values < 0.05 were considered significant, highlighted by numbers in bold print

#### Clinical presentations

The time between SARS-CoV-2 infection and onset of movement disorders, cerebellar or pyramidal signs was heterogeneous, ranging from 0 to 280 days with a median of 3 days. 79% (26/33) of the children predominantly presented with cerebellar signs [5, 6, 20–22, 26–33]. 21% (7/33) were described with hypo- or hyperkinetic movement disorders (see “Results 5, 6”, Fig. 2**, **Table 3) [5, 23–25, 34], and no patient showed predominant parkinsonism. In three cases, including our study, videos for the exact description of movement disorder phenomenology were provided [26, 33]. Overall, neuropsychiatric symptoms such as confusion, irritability, headache, and cognitive impairment were described in 60% (18/30) of reported cases [6, 20–25, 27, 29–33]. The criteria for MIS-C after COVID-19 infection, according to the definition of the World Health Organization (WHO) [19], was met in 21% of cases (7/33) with a median age of 6 years (range 1.4–15.0 years, clinical signs: ataxia, dysarthria, gait instability).

#### Clinical investigations

70% (19/27) of patients had a positive nasopharyngeal SARS-CoV-2 RT-qPCR on admission [6, 20, 22, 24, 25, 27–31, 33, 34]. In 86% of cases (24/28), clinical investigations revealed pathological findings and only in 14% (4/28), no abnormalities were found neither in sera, nor CSF, brain MRI, or EEG [6, 24, 26]. Brain MRI results were abnormal in 53% (16/30) [6, 20–22, 25, 27, 31, 32] and showed cerebellar signal alterations in 31% (5/16), and white matter changes in 31% (5/16), of which three MRI met criteria of acute demyelinating encephalomyelitis (ADEM) (Supplementary table 1). One patient (6%) was diagnosed with Guillain-Barré syndrome and correspondingly presented with thickening of nerve roots. Another patient showed typical signs of Wernicke encephalitis on MRI. In 25% (4/16), neuroimaging revealed signal alterations in the corpus callosum of unknown significance. EEG was performed in 33% (11/33) of cases only. Pathological findings were detected in 6 out of 11 [20, 21, 25, 27, 30]; 4 of these 6 patients had been diagnosed with MIS-C. Concerning CSF findings, SARS-CoV-2 RT-qPCR was performed on eight samples, yielding negative results in all cases [20, 21, 25, 28, 29, 31, 33]. Criteria for inflammatory CSF, broadly defined as pleocytosis or elevated protein level or positive oligoclonal bands or CSF anti-neuronal/-glial antibodies or positive immunofluorescence on murine brain sections, were met in 64% (16/25) [6, 20, 23, 25, 27–31, 33]. (1) Here, protein levels and white blood cell counts were only slightly elevated in comparison to normal ranges, with a median of 0.48 g/l (range 0.08–19.0 g/l) and a median of 5 cells/µl (range 1–2000/µl), respectively. (2) CSF antibodies against SARS-CoV-2 could be detected in two of three reported cases [23, 33]. (3) Screening for anti-neuronal/-glial antibodies in CSF was carried out in ten cases. Three of those ten samples revealed positive results (see “Results 5”). (4) In two children, including the index patient described in this study, elevated CSF protein levels but negative panels for anti-neuronal/-glial antibodies led to additional immunofluorescence studies via tissue-based assays on murine brain (see “Results 6”).

#### Treatment and outcome

Treatment and outcome were reported in 28/33 cases. In 14% (4/28) of patients, the symptoms resolved spontaneously, and treatment was not necessary [6, 20, 28, 29]. One patient with thiamine deficiency and cerebellitis was supplemented with thiamine [32] and another patient received carbamazepine to reduce choreatic movements of a suspected Sydenham’s chorea relapse [34]. The other 79% (22/28) of children were treated with immunotherapy. Treatment with steroids only was sufficient in 21% (6/28), with five patients receiving high-dose methylprednisolone intravenously and one patient being treated with oral prednisone [6, 33]. IVIG as a monotherapy were administered in one child with Guillain-Barré syndrome [25]. 36% (10/28) of children received a combined therapy of methylprednisolone and IVIG, of which three studies reported a better response to methylprednisolone including our case report [6, 26]. Another 18% (5/28) of children needed therapy escalation (e.g. anakinra, rituximab, plasma exchange). The majority of patients (57%, 16/28) fully recovered after immunotherapy and in approximately one third (29%, 8/28), a partial recovery after treatment was described. Overall, no relapses were reported within a relatively short observation time of two months (median; range 0.25–6 months).

We investigated whether there were differences in the outcome depending on the phenomenology or the severity of symptoms (Supplementary table 2). In children with cerebellar signs, 17% (4/23) recovered spontaneously, 61% (14/23) recovered fully after treatment and 22% (5/23) recovered only partially after treatment. In children with hypo-/hyperkinetic movement disorders, none recovered spontaneously, 40% (2/5) recovered fully after treatment and 60% (3/5) recovered only partially after treatment. In the MIS-C group, one child (25%) recovered spontaneously, two (50%) after treatment and one (25%) only partially after treatment.

We additionally compared outcomes depending on the clinical investigations (Supplementary table 2): all four patients without pathological diagnostic findings were treated with immunotherapy and reached full recovery after one month. There was a trend of better outcome in patients without inflammatory CSF in comparison to patients with inflammatory CSF: 89% (8/9) *versus* 75% (12/16) recovery completely spontaneously/after treatment, and 11% (1/9) *versus* 25% (4/16) recovered partially after treatment. Brain MRI with or without pathological findings did not seem to change the outcome: 75% recovered completely spontaneously/after treatment, and 25% recovered partially after treatment. EEG was performed in a limited number of patients only.

#### Patients with COVID-19-associated ataxia/cerebellitis

79% (26/33) of the children presented predominantly with cerebellar signs (Fig. 2, Table 3) [5, 6, 20–22, 26–33]. The number of patients with positive respiratory SARS-CoV-2 RT-qPCR on admission was 80% (16/20). Correspondingly, patients with proven SARS-CoV-2 seroconversion were less frequent with 20% (4/20). Children were on average nine years old (median; range 0.1–15.3 years). The onset of cerebellar symptoms was usually immediately after infection (median of 0 days; range 0–280 days). The majority of cases (24/26) presented with ataxia. Within this group, from the clinical description published, we were able to assign the diagnosis of acute cerebellar ataxia (ACA) [6, 28, 29] in 12% (3/26) of patients and acute cerebellitis (AC) [6, 20, 21, 27, 30–33] in 54% (14/26), according to respective definitions (for detailed comparison see Supplementary table 3) [35]. Additionally, three patients with ataxia had been diagnosed with ADEM [6], one 7-year-old boy with anti-*N*-methyl-d-aspartate receptor (NMDAR) encephalitis [30], one 9-year-old boy with Guillain–Barré syndrome [22], one 14-year-old girl with thiamine deficiency [32], and three cases were not described in detail [5]. Neuroimaging revealed pathologic results in 65% (15/23) and EEG was pathologic in four out of four reported cases. Criteria of inflammatory CSF were met in 62% (13/21). Aubart et al*.* described two patients with ataxia, who were tested positive for anti-myelin oligodendrocyte glycoprotein (MOG) antibodies and showed signs of ADEM on brain MRI [6]. Sarigecili et al*.* reported on one patient with anti-NMDAR antibodies in CSF with predominant cerebellar signs and choreatic movements in the course of disease [30]. In children with COVID-19-associated ataxia/cerebellitis, 71% (18/23) recovered completely spontaneously/after treatment, and 22% (5/23) recovered only partially after treatment.

#### Patients with COVID-19-associated hypo-/hyperkinetic movement disorders

21% (7/33) of patients were described with hypo-/hyperkinetic movement disorders (Fig. 2, Table 3) [5, 23–25, 34]. 43% (3/7) of patients had a positive respiratory SARS-CoV-2 RT-qPCR on admission. In 57% (4/7), seroconversion could be verified. Children were on average 13 years old (median; range 9.0–16.0 years). Movement disorders evolved with a median delay of 3 days (range 3–80 days) after infection. Within this group, five of seven children were described with chorea [5, 23, 34], which was mainly left-sided in two cases [34]. One of those patients were suspected with a relapse of Sydenham’s chorea [34]. Additionally, Della Corte et al*.* reported on one 12-year-old boy with left-sided myoclonus, bilateral clonus (pyramidal sign), and psychiatric symptoms [24]. Gaughan et al*.* described a 16-year-old girl with acute onset akinesia interpreted as akinetic mutism, rigidity and potentially iatrogenic tremor after treatment with haloperidol and olanzapine [25]. In patients with chorea, neuroimaging (0/5) and EEG (0/3) did not revealed any pathological results. Criteria of inflammatory CSF were met in two out of two cases. In two children, including our index patient, elevated CSF protein levels but negative results of panel testing for anti-neuronal/-glial antibodies led to additional immunofluorescence studies via tissue-based assays on murine brain sections. Bartley et al*.* identified positive immunosignals in mitral cells of the olfactory bulb, cerebral cortex, cerebellar Purkinje cells, hippocampus, and brainstem of a mouse brain (with no information on the basal ganglia) with CSF from a mid-teen girl with chorea and psychiatric impairment [23]. Whereas, in our case, an isolated astrocytic fiber pattern could be detected in the murine hippocampus, basal ganglia, and cerebellum. In children with chorea, none of three reported cases recovered spontaneously, one recovered fully and two others only partially after treatment.

## Discussion

The COVID-19 pandemic is ongoing and while mortality rates are decreasing, diagnosis and treatment of neurologic disease associated to SARS-CoV-2 infection remains a challenge for pediatricians [7]. A prospective cohort study including children with COVID-19 hospitalized in England between 2020 and 2021 estimated a prevalence of 3.8 per 100 pediatric cases with neurologic and psychiatric impairment [5]. One-tenth of those cases (5/51) had movement disorders (chorea) or cerebellar impairment (ataxia). Applying these epidemiologic data to 14 million children having been tested positive for COVID-19 by February 2022 in the United States [2], approximately 50 thousand children would be affected of non-functional de novo movement disorders or ataxia. Additionally, a rising incidence of functional movement disorders during the COVID-19 pandemic was recently reported [10]. To facilitate differentiation and improve care, it is necessary to provide missing data on the phenomenology, clinical findings, treatment options and outcome of non-functional pediatric de novo movement disorders, cerebellar and pyramidal signs associated to SARS-CoV-2 infection or vaccination. We performed a scoping review from 2019 to 2022 on MEDLINE and identified 17 studies describing 32 pediatric cases of de novo movement disorders, cerebellar or pyramidal signs after SARS-CoV-2 infection. Additionally, we reported on a 10-year-old girl with hemichorea after SARS-CoV-2 infection/vaccination.

**Fig. 3:**
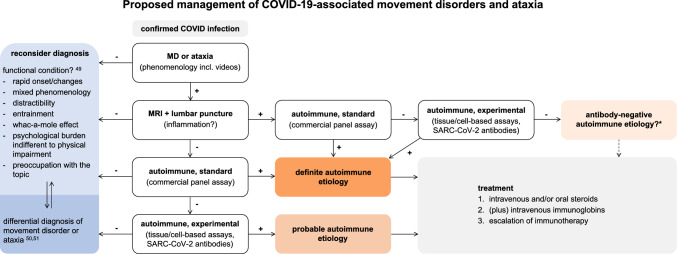
A diagnostic and therapeutic algorithm for the management of COVID-19-associated movement disorders and ataxia. This proposed algorithm is derived from the here published data (retrospective, *n* = 33) and is therefore of advisory nature. For differential diagnoses please refer to the recommendations for clinical use of the European Reference Network Rare Neurological Disease (ERN-RND) for patients with “childhood-onset chorea” and “early-onset ataxias” and the review on functional movement disorders by Edwards and Bhatia [49–51]. The terms “probable” and “definite autoimmune etiology” are based on the definitions for autoimmune encephalitis [52]. Details on tissue- and cell-based assays, as well as an example of a commercial panel assay for anti-neuronal antibodies are stated in the Supplementary methods 1. *In cases of suspected autoimmune etiology but absence of autoantibodies, consider antibody-negative autoimmune encephalitis following published criteria [52] and discuss probatory immunotherapy after exclusion of alternative causes. *MD* movement disorders, *MRI*: magnetic resonance imaging

SARS-CoV-2-associated pediatric movement disorders or cerebellar impairment occurred in a minority in the context of known entities such as Guillain-Barré syndrome [22], Sydenham’s chorea [34], thiamine deficiency [32], ADEM [6], or encephalitis with known antibodies (NMDAR, MOG) [6, 30]. A larger proportion, however, could not be assigned to a defined clinical entity. Based on the published data, we propose a diagnostic work-up for differential diagnosis and therapeutic algorithm for the management of COVID-19-associated movement disorders and ataxia in children (Fig. 3).

It appears that in most pediatric cases of COVID-19, two different neurological systems may be affected: (i) the cerebellar system and (ii) the cortico-basal ganglia network.

(ad i) Both, acute cerebellar ataxia (ACA) and acute cerebellitis (AC) are well-known (post)infectious conditions in children, with ACA being the most common cause for acute ataxia in pediatric emergency departments [36]. Whereas these patients typically present with chief complaints as ataxia, unsteady gait, nystagmus, or dysmetria, children with AC additionally suffer of altered mental state and more frequently show brain MRI abnormalities [35]. Children with ACA are typically under the age of 6 years and the outcome is very good. Segal et al*.* reported on a cohort of 58 children, 72% of whom made a complete spontaneous recovery within 1–2 days and the remainder within 3–21 days. [17] Yet, after AC, Lancella et al*.* reported on neurologic sequelae in 6% of children, and those with pathologic findings on neuroimaging were more likely to have only partial recovery. In our cohort, we describe 26 children with predominant cerebellar signs in the context of COVID-19 (Fig. 2, Table 3, Supplementary Table 3). Only 12% could be classified as ACA, whereas most patients (54%) met criteria of AC. Chief complaints were ataxia, unsteady gait or dysmetria; and in many cases (70%) abnormalities on brain MRI were identified. Unlike ACA/AC, the median age of these patients with COVID-19-associated ataxia/cerebellitis was higher at 9 years and the onset of ataxia was earlier, with 70% of the children developing movement disorders during the acute course of infection. The outcome of these children was less favorable in comparison to ACA/AC: only 17% recovered without treatment, another 61% recovered completely after therapy, and 22% of patients recovered only partially after treatment. Within the small subgroup of three cases meeting the criteria of ACA, the outcome was better with 67% of spontaneous recovery and 33% fully recovering after treatment. To confirm these two entities in de novo ataxia/cerebellitis after SARS-CoV-2 infection, and to further identify prognostic parameters and long-term outcomes, larger cohorts and longer follow-up periods will be necessary. Even so, COVID-19-associated ataxia/cerebellitis seems to have distinct characteristics and immunotherapy may be superior to watchful waiting.

(ad ii) The basal ganglia play a vital role in initiating and controlling movement and behavior. Autoimmune processes in this finely tuned network can trigger hypo- and hyperkinetic movement disorders [37, 38]. After streptococcal infection, e.g., prototypic chorea and psychiatric symptoms may evolve, known as Sydenham’s chorea [18]. Dale et al*.* identified antibodies against surface dopamine-2 receptor in 10/30 patients with Sydenham’s chorea [37], indicating a post-infectious humoral autoimmunity. Yet, much of the pathophysiology remains unclear. The treatment regimens are diverse, with approximately one-third of children receiving immunotherapy. The outcome is favorable with remission in most cases [18]. We found a very limited number of patients with hypo-/hyperkinetic movement disorders after SARS-CoV-2 infection (Fig. 2**, **Table 3). It may be considered that hyperkinetic movement disorders can be mistaken as “ataxia” by the untrained eye and videos were rarely provided. Considering the limited number of cases, none of five patients with chorea had abnormal brain MRI or EEG. Two patients met the criteria for inflammatory CSF with elevated protein levels, but both screening was negative for anti-neuronal/-glial antibodies: [39] In one mid-teen patient with chorea and psychiatric symptoms, both CSF antibodies against SARS-CoV-2 of unknown significance and a positive result in immunofluorescence testing on murine brain (with no information on the basal ganglia) were detected [23]. In a 10-year-old girl with left-sided hemichorea (our index patient), a likely autoimmune astrocytic fiber pattern in the murine *hippocampus*, basal ganglia, and *cerebellum* was found. In both cases, the exact target of these autoantibodies binding to neuronal or glial surface receptors or synaptic proteins, was not identified. Yet, both patients benefited from immunotherapy. The overall outcome of COVID-19-associated chorea seemed to be less favorable in comparison to Sydenham’s chorea with 67% of only partial recovery after therapy. Interestingly, two other patients with chorea and one with myoclonus were described with striking left-sided predominance, which is a well-known phenomenon in immune-mediated movement disorders [18, 24, 34]. To date, the molecular mechanism is unclear. There are only hints suggesting that asymmetrically expressed proteins may be targets of autoantibodies [40] or that a circulating pathogenic B-cell clone releasing autoantibodies passes the blood–brain barrier only on one hemisphere. [41]

The relevance of post-viral humoral autoimmunity in the disease cascade of COVID-19 is emphasized by emerging reports of anti-neuronal and anti-glial antibodies in CSF of patients with neurologic symptoms [13, 14]. In this study, SARS-CoV-2 virus was not detected in the CSF of any of the patients tested. Instead, the presence of autoantibodies is supported by both, unspecific indirect immunofluorescence techniques and hypothesis-driven cell-based assays. The exact identification of those antibodies is difficult. The response to immune-modulating therapy, however, suggests an autoimmune process in the pathophysiology of COVID-19-associated de novo movement disorders or cerebellar involvement [42]. Results of this study indicate a better responsiveness to high-dose intravenous methylprednisolone, which we take into account in our algorithm (Fig. 3).

Possibilities for further and more granular analysis were limited as the total number of patients was small, sample sizes varied widely between specific disease groups, and the data might be susceptible to reporting bias due to the preferential publication of severe and special cases. In particular, the comparative group analysis performed to identify potential prognostic factors (Supplementary table 2) must be interpreted with caution, because of the limitations mentioned above. Yet, our data (Table 3) show a trend of good responsiveness to immune-modulating therapy, especially steroids, and of better outcomes in the absence of inflammatory CSF.

In contrast to a comprehensive review about de novo movement disorders in an adult cohort of 52 patients [12], no parkinsonism, and fewer cases with myoclonus were seen in our pediatric cohort. Cerebellar signs were similarly described in the adult cohort, whereas chorea was less common in adult patients [12, 43]. In adults, three cases were published with acute hemichorea after vaccination with AstraZeneca® or Comirnaty® [44, 45]. Of note, only one child in our study developed movement disorders after COVID-19 vaccination, although even in this case, the preceding COVID-19 infection cannot be excluded as the actual trigger. We rather assumed that mechanisms of post-viral humoral autoimmunity may also be triggered by vaccination, but more mildly in comparison to infection. A protective effect of COVID-19 vaccinations to prevent MIS-C could already be shown in a Danish cohort [46].

## Conclusion

Infection with SARS-CoV-2 can trigger de novo movement disorders and cerebellar impairment in children and adolescents, likely through post-viral humoral autoimmunity. Most children presented with signs of COVID-19-associated ataxia comprising distinct characteristics different from other post-infectious ataxias or cerebellitis (older age, less favorable outcome). Fewer cases presented with chorea. Here, we identified CSF antibodies against structures of the basal ganglia, but details on the pathophysiology are yet to be discovered. Our data suggest that children with de novo movement disorders, and cerebellar inflammation can benefit from immune-modulating therapy, especially methylprednisolone, despite negative results in clinical investigations. Larger case numbers and longer clinical follow-up investigations will be necessary to further unravel the spectrum of pediatric COVID-19-associated ataxia/cerebellitis and COVID-19-associated chorea and potentially confirm these new clinical entities.

### Supplementary Information

Below is the link to the electronic supplementary material.

Supplementary file1 Video 1: Ten-year-old girl with COVID-19-associated hemichorea before treatment. Neurological examination of a ten-year-old girl with striking hemichorea associated to SARS-CoV-2 infection and vaccination (MOV 14447 KB)

Supplementary file2 Video 2: Ten-year-old girl with COVID-19-associated hemichorea after intravenous immunoglobulin (IVIG) therapy. Neurological examination of the patient two days after IVIG therapy for two days (1000 mg/kg/d). The UFMG Sydenham’s Chorea Rating Scale (USCRS) score had slightly improved from 26 to 20. However, 13 days after IVIG treatment the score had worsened again to 31 (MOV 8190 KB)

Supplementary file3 Video 3: Ten-year-old girl with COVID-19-associated hemichorea one month after high-dose intravenous methylprednisolone (MPS) therapy. Neurological examination of the patient five days after MPS therapy for five days (20 mg/kg/d). The UFMG Sydenham’s Chorea Rating Scale (USCRS) score had drastically improved from 31 to 3 (MOV 7606 KB)

Supplementary file4 (XLSX 16 KB)

Supplementary file5 (XLSX 11 KB)

Supplementary file6 (XLSX 12 KB)

Supplementary file7 (DOCX 23 KB)

## Data Availability

Data are available upon reasonable request.
