# Supplementary material for: Pediatric de novo movement disorders and ataxia in the context of SARS-CoV-2
Source: J Neurol. 2023 Jul 29;270(10):4593–607. doi: 10.1007/s00415-023-11853-5 (PMC10511612; doi:10.1007/s00415-023-11853-5)
Supplement: Supplementary file 7 — Supplementary file7 (DOCX 23 KB) [file 415_2023_11853_MOESM7_ESM.docx]

**Supplementary methods 1**

**Case report**

**1 Clinical description**

Data on the clinical presentation, diagnostic work-up, treatment and outcome were collected retrospectively. The movement disorder was evaluated in the outpatient clinic by a first physician, an occupational therapist, a physiotherapist and independently by a second physician through standardized video recordings. The severity of chorea was assessed with the UFMG Sydenham’s Chorea Rating Scale (USCRS) (Teixeira *et al.*, Mov Disord, 2005). SARS-CoV-2 RT-qPCR testing was done by RT-qPCR from nasopharyngeal swabs and CSF after lumbar puncture. Cerebral magnetic resonance images (MRI) and electroencephalogram (EEG) were recorded. Lumbar puncture and measurement of the opening pressure were done in the lying position. In serum and CSF, we tested for SARS-CoV-2 IgG antibodies against the nucleocapsid and the spike protein. Ophthalmological examinations comprised fundoscopy and optical coherence tomography (OCT). Whole exome sequencing of the patient’s DNA was done commercially (CeGaT GmbH, Tübingen, Germany).

**2 Screening for the presence of antineuronal antibodies**

Patient CSF and serum had been analyzed during clinical work-up by a commercial panel assay (“Biochip Mosaic”, EUROIMMUN Clinical Immunological Laboratory, Lübeck, Germany) for known surface and paraneoplastic antibodies including antibodies against AMPAR1, AMPAR2, AT1A3, cANCA, amphiphysin, aquaporin-4, CASPR2, CENP-B, CNTN1, CV2, dopamine-2 R, DPPX, dsDNA, ERC1, GABAA R, GABAB R, GAD65, GluRD2, GlyR, GFAP, Homer 3, Hu, Jo-1, KCNA2, La, LGl1, Ma2/Ta, mGluR5, MOG, myelin, neurexin-3-alpha, neurochondrin, neurofascin NF155, NF166, NMDAR, pANCA, rhoGTPase-activating protein 26, Ri, RNP-70, Ro, Scl-70, Sm, Tr/DNER, U1RNP, Yo. In addition, serum was tested for anti-MOG IgG *via* live cell-based assay (CBA; Dr. F. Leypoldt, Neuroimmunology, Institute of Clinical Chemistry, and Department of Neurology, University Medical Center Schleswig-Holstein, Kiel, Germany).

**3 Immunological studies with patient CSF and serum on mouse brain tissue and in cell culture**

In addition to the clinical routine, we performed a tissue-based assay (TBA) according to established protocols. (Nikolaus *et al*., Eur J Paediatr Neurol, 2020; Kreye *et al*., Brain J Neurol., 2016). For immunostaining, we used both unfixed and 4% PFA-fixed murine brain sections (animal experimentation registration number LaGeSo Berlin T0118/17). The tissue was incubated with either undiluted CSF or serum at a 1:100 dilution. A human recombinant monoclonal antibody without neuroreactivity served as negative control. Serum of a healthy individual served as background control. A monoclonal anti-NMDAR antibody served as positive control. For the cell-based assay (CBA), HEK293T cells were transiently transfected with GFAP DNA (1 μg). Staining procedure and imaging were performed as previously described^20,21^ but using a THUNDER Imager DMi8 with a Leica DFC9000 GT camera (Leica) and corresponding LAS(X) software.
